# How to Process Sputum Samples and Extract Bacterial DNA for Microbiota Analysis

**DOI:** 10.3390/ijms19103256

**Published:** 2018-10-20

**Authors:** Leonardo Terranova, Martina Oriano, Antonio Teri, Luca Ruggiero, Camilla Tafuro, Paola Marchisio, Andrea Gramegna, Martina Contarini, Elisa Franceschi, Samantha Sottotetti, Lisa Cariani, Annamaria Bevivino, James D. Chalmers, Stefano Aliberti, Francesco Blasi

**Affiliations:** 1 Fondazione IRCCS Cà Granda Ospedale Maggiore Policlinico, Pediatric Highly Intensive Care Unit, 20100 Milan, Italy; leonardo.terranova@policlinico.mi.it (L.T.); luca.ruggiero@policlinico.mi.it (L.R.); camilla.tafuro@policlinico.mi.it (C.T.); paola.marchisio@unimi.it (P.M.); 2 Department of Clinical Sciences and Community Health, University of Milan, 20100 Milan, Italy; 3 Department of Pathophysiology and Transplantation, University of Milan, Internal Medicine Department, Respiratory unit and Adult Cystic Fibrosis Center, Fondazione IRCCS Ca’ Granda Ospedale Maggiore Policlinico, 20100 Milan, Italy; martina.oriano@unimi.it (M.O.); gramegna.med@gmail.com (A.G.); contarini.martina@gmail.com (M.C.); elisa.franceschi.93@gmail.com (E.F.); francesco.blasi@unimi.it (F.B.); 4 Department of Molecular Medicine, University of Pavia, 27100 Pavia, Italy; 5 Cystic Fibrosis Microbiology Laboratory, Fondazione IRCCS Ca’ Granda, 20100 Milan, Italy; antonioteri@hotmail.it (A.T.); samantha.sottotetti@hotmail.it (S.S.); lisa.cariani@gmail.com (L.C.); 6 Department for Sustainability, Italian National Agency for New Technologies, Energy and Sustainable Economic Development (ENEA), 00123 Rome, Italy; annamaria.bevivino@gmail.com; 7 Scottish Centre for Respiratory Research, University of Dundee, Ninewells Hospital and Medical School, DD1 9SY Dundee, UK; j.chalmers@dundee.ac.uk

**Keywords:** DNA extraction, sputum, microbiota, sequencing

## Abstract

Different steps and conditions for DNA extraction for microbiota analysis in sputum have been reported in the literature. We aimed at testing both dithiothreitol (DTT) and enzymatic treatments of sputum samples and identifying the most suitable DNA extraction technique for the microbiota analysis of sputum. Sputum treatments with and without DTT were compared in terms of their median levels and the coefficient of variation between replicates of both DNA extraction yield and real-time PCR for the 16S rRNA gene. Treatments with and without lysozyme and lysostaphin were compared in terms of their median levels of real-time PCR for *S. aureus*. Two enzyme-based and three beads-based techniques for DNA extraction were compared in terms of their DNA extraction yield, real-time PCR for the 16S rRNA gene and microbiota analysis. DTT treatment decreased the coefficient of variation between replicates of both DNA extraction yield and real-time PCR. Lysostaphin (either 0.18 or 0.36 mg/mL) and lysozyme treatments increased *S. aureus* detection. One enzyme-based kit offered the highest DNA yield and 16S rRNA gene real-time PCR with no significant differences in terms of alpha-diversity indexes. A condition using both DTT and lysostaphin/lysozyme treatments along with an enzymatic kit seems to be preferred for the microbiota analysis of sputum samples.

## 1. Introduction

The study of the human microbiome has emerged as an important field in translational research over the past decade, and promising applications of this technique in clinical practice have been suggested [1]. Most of the recent advances in microbial ecology have been made possible thanks to the development of next-generation sequencing technologies which allow the analysis of complex microbial populations in different body niches, including the respiratory tract. Multiple studies of the human-associated microbiota have suggested a fundamental role of microbes, and predominantly bacteria, in maintaining health and a role for changes in lung microbiota in contributing to diseases such as chronic obstructive pulmonary disease (COPD), bronchiectasis and cystic fibrosis (CF) [2,3,4,5,6,7].

Most of the published literature evaluating microbiota in chronic respiratory infections has used sputum as the preferred sample type. Although potentially less representative of the deep lung as compared with bronchoalveolar lavage (BAL), sputum could represent the preferred non-invasive diagnostic technique to be used in clinical practice for microbiota analysis. However, metagenomic sequencing from sputum is challenging because of the complex matrix of sputum, its heterogeneity, the activity of proteases and nucleases and the variability in sputum quality. Little has been published regarding the optimal methods for nucleic acid extraction from sputum for use in microbiome studies. Some bacteria require high levels of mechanical disruption or enzymatic disruption in order to achieve efficient lysis, and these include important pathogens in bronchiectasis and cystic fibrosis such as *Staphylococcus aureus*. [8,9].

Different techniques have been used so far for microbiota analysis in respiratory samples, resulting in heterogeneity in terms of sample processing for DNA extraction, library preparation and bioinformatics analysis [4,10]. With a view to being able to compare the results of respiratory microbiota studies from different cohorts and different diseases, this heterogeneity needs to be approached, and optimal operating procedures to evaluate microbiota from sputum should be identified. Several steps should be standardized, including the use or not of fluidifying agents, such as dithiothreitol (DTT), or enzymes for specific bacterial lysis, such as lysozyme and lysostaphin for *S. aureus*, as well as different methods for DNA extraction and isolation. Although a comparison between different methods for DNA extraction in both BAL and gastrointestinal samples has already been carried out, no previous studies have specifically addressed this topic for sputum samples [11,12].

The present study had three objectives: (1) to evaluate the performance of DTT treatment to homogenize sputum samples; (2) to evaluate the effect of an enzymatic step for Gram-positive lysis; and (3) to investigate the performance of lytic chemical/enzyme-based and bead-based extraction kits.

## 2. Results

**Study patients.** A total of 32 adult patients with CF and bronchiectasis were enrolled in this study, and 77 sputum samples were collected for the three experiments.

**Figure 1:**
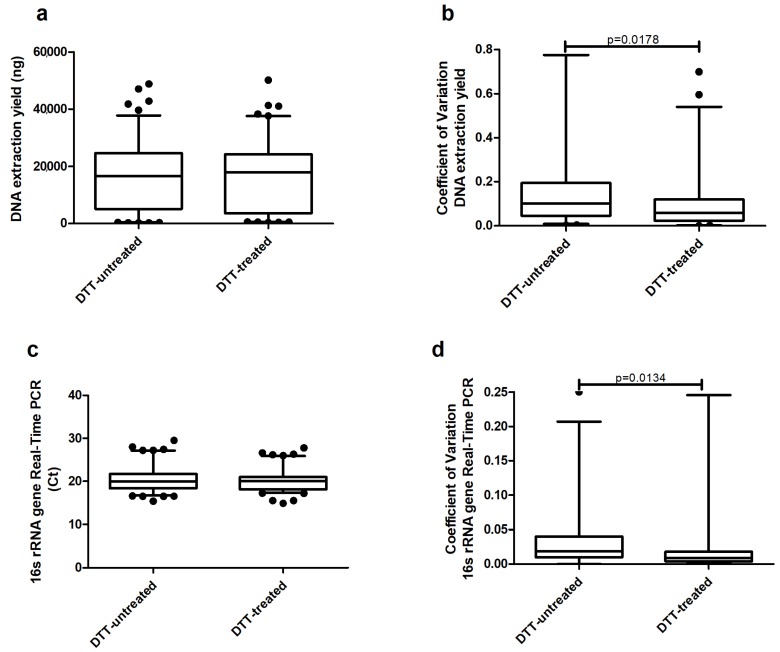
Comparison of median levels of (**a**) DNA extraction yield, (**b**) coefficient of variation between replicates, (**c**) 16s rRNA gene real-time PCR results, and (**d**) coefficient of variation between replicates across dithiothreitol (DTT)-untreated sputum vs. DTT-treated (Experiment 1).

**Experiment 1—DTT treatment evaluation.** The median values of extracted DNA were similar between the DTT-untreated and DTT-treated group (16.630 vs. 17.880 μg, *p* = 0.7211) (Figure 1a). The coefficient of variation between replicates was statistically lower in sputa treated with DTT in comparison with not-treated aliquots (DTT-untreated 0.1014 vs. DTT-treated: 0.0592, *p* = 0.018), (Figure 1b). A similar situation has been identified when the analysis of the 16s real-time PCR was performed (Figure 1c,d). Although no differences were detected between groups in terms of their median *C*t (DTT-untreated: 19.93 vs. DTT-treated: 20.09, *p* = 0.519), there was a statistically significant decrease of the coefficient of variation in the DTT group compared to the not-treated one (DTT-untreated: 0.01859 vs. DTT-treated: 0.008848, *p* = 0.013).

**Figure 2:**
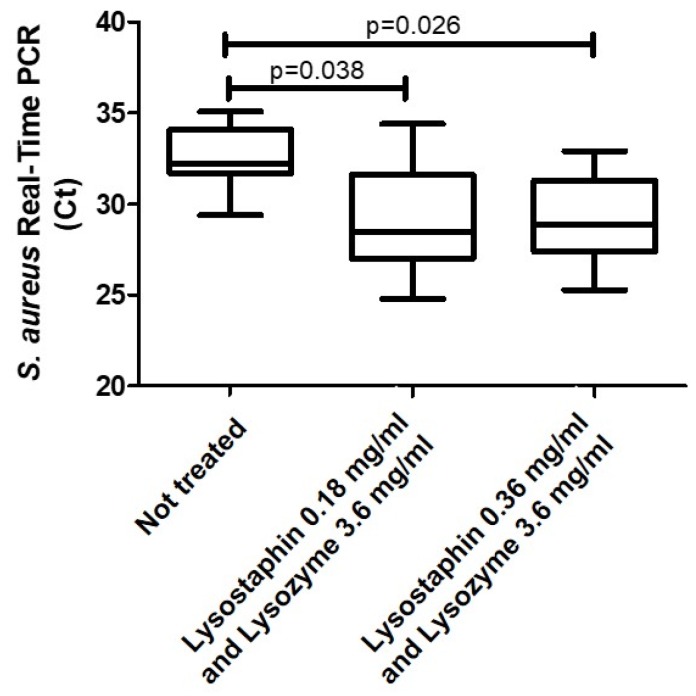
Comparison of median levels of real-time PCR for *S. aureus* across samples treated with lysostaphin at either 0.18 or 0.36 mg/mL and lysozyme (3.6 mg/mL) or without lytic enzymes treatment. Results expressed in cycle threshold (*C*t). Kruskal–Wallis test: *p* = 0.041 (Experiment 2).

**Experiment 2—Lytic enzymes evaluation.***S. aureus* was detected from all the evaluated samples. Significantly high bacterial loads of *S. aureus*, represented by significantly lower median *C*t levels by real-time polymerase chain reaction (PCR), were identified in samples treated with lytic enzymes compared with the non-treated ones (*p* = 0.038 for lysostaphin 0.18 mg/mL and lysozyme 3.6 mg/mL; *p* = 0.027 for lysostaphin 0.36 mg/mL and lysozyme 3.6 mg/mL). No significant difference was identified between the median levels of *C*t among samples treated with the two dosages of lysostaphin and lysozyme (*p* = 1) (Figure 2).

**Figure 3:**
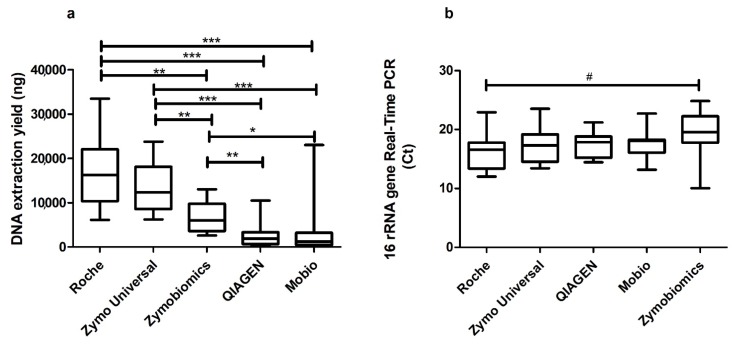
Comparison of median levels of (**a**) DNA extraction yield and (**b**) bacterial extraction efficiency across the five analyzed commercial kits. Kruskal–Wallis test: DNA extraction *p* < 0.0001; 16s rRNA gene real-time PCR *p* = 0.169. Mann–-Whitney test: *: *p* ≤ 0.001; **: *p* ≤ 0.0009, *** *p* ≤ 0.0001; #: *p* = 0.027 (Experiment 3).

**Experiment 3—Evaluation of different kits for bacterial DNA extraction.** DNA extraction yield reached the highest values using the Roche kit in comparison to all the other kits. The median (interquartile range, IQR) yields for DNA extracted with the five kits were as follows: Roche 16,250 ng (6100–33,500 ng); Zymo Universal 12,350 ng (6225–23,750 ng); Zymobiomics 5975 ng (2625–13,000 ng); QIAGEN 1913 ng (204.5–10,475 ng); and Mobio 1205 ng (4.1–23,000 ng). Significant differences were found across the five kits (*p* < 0.0001) and between two or more kits (Figure 3). No statistical differences were found between Roche and Zymo Universal in terms of DNA recovery (*p* = 0.3506).

Results from the 16s rRNA gene Real-Time PCR showed a similar median *C*t across the five extraction methods (*p* = 0.169). The median (IQR) Ct values were as follows: Roche 16.57 (12.01–22.94); Zymo Universal 17.29 (13.42–23.55); QIAGEN 17.86 (14.45–21.22); Mobio 18.87 (13.16–22.72); and Zymobiomics 19.57 (10.04–24.85). The only significant difference was found between *C*t values obtained using the Roche and Zymobiomics kits (*p* = 0.027).

A low number of joined reads (min 47–max 253) was produced for each negative control and no bacteria were identified. None of the operational taxonomic units (OTUs) assigned to these samples passed the 0.5% prevalence threshold that was set to filter out low-abundance OTUs from the OTU table considered to be due to cross-talk or experimental error.

**Figure 4:**
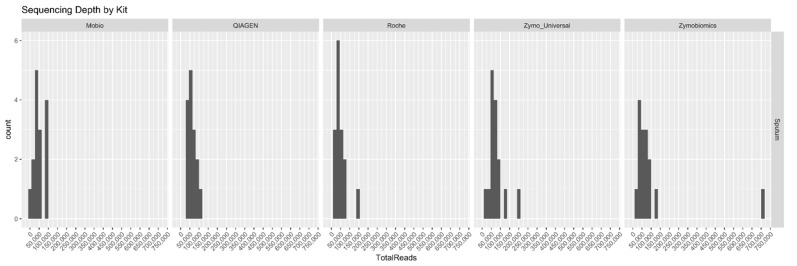
Sequencing depth of samples by kit.

The sequencing depth was nearly the same for all samples which were extracted with the different kits (typical number of reads comprised between 0 and 100,000), except for one. One sample extracted with Zymobiomics was sequenced ~ 7 times more deeply than all the others (Figure 4).

The only sample we were not able to sequence belonged to the Mobio group.

**Figure 5:**
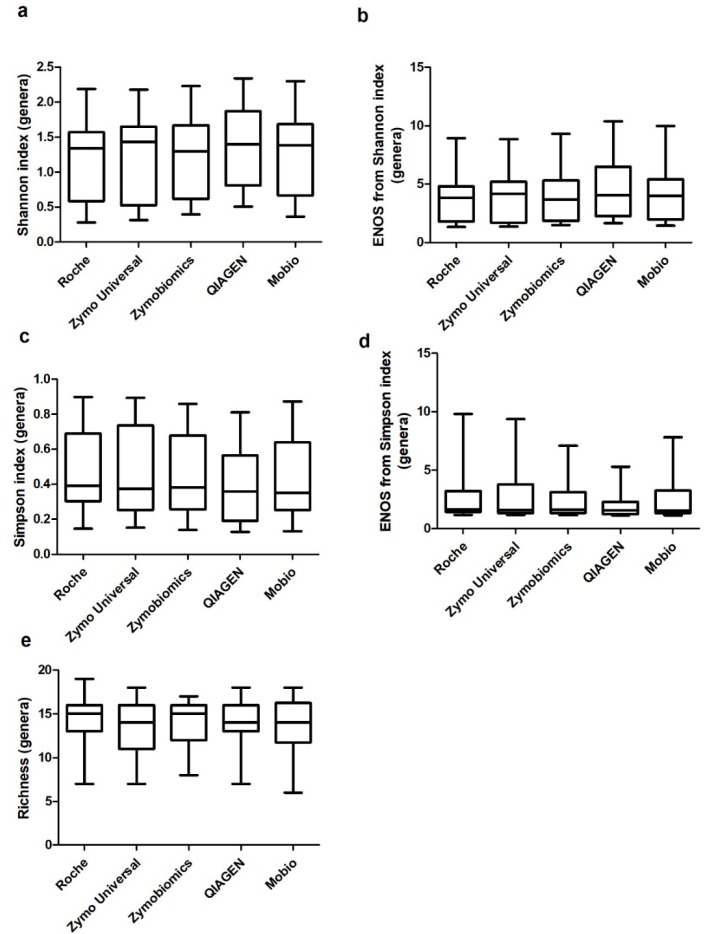
Comparison of median levels of (**a**) the Shannon index, (**b**) effective number of species (ENOS) from Shannon, (**c**) the Simpson index, (**d**) ENOS from the Simpson index, and (**e**) richness across the five analyzed commercial kits (Experiment 3).

None of the tested techniques showed a statistical difference compared to the others in any of the considered alpha indices from genera (Figure 5). 

**Figure 6:**
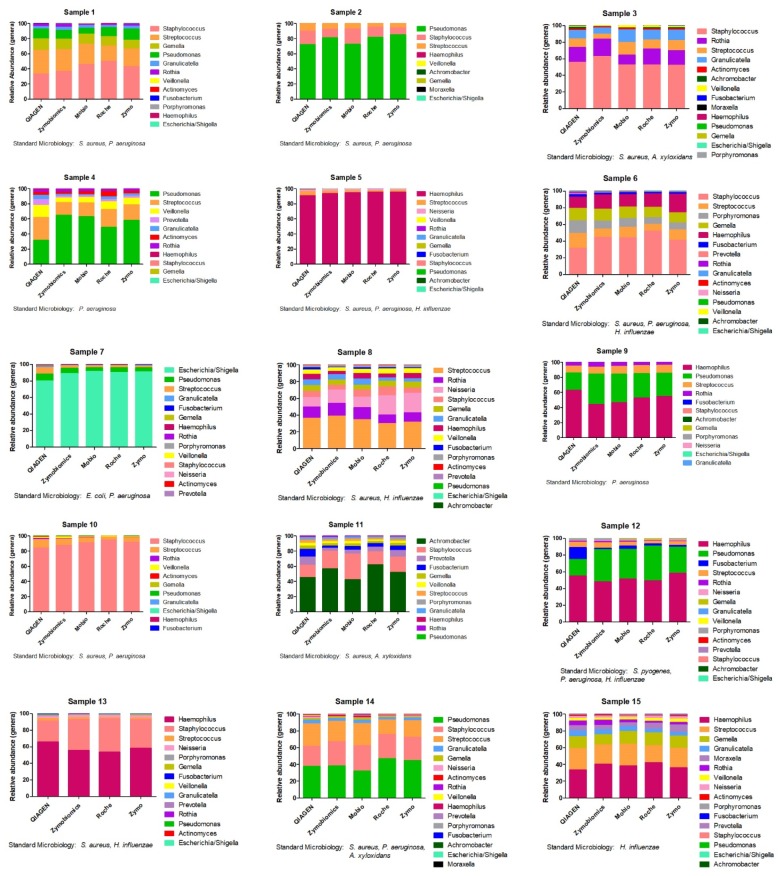
Genera-relative abundances of 15 patients for each of the considered methods (Experiment 3).

The relative abundances of the 15 samples were measured using the five evaluated kits and are presented in Figure 6 along with the results of cultures from standard microbiology. The comparison between genera retrieved through 16S rRNA gene sequencing and culture results showed a complete concordance of the two techniques at the genus level, even if, as expected, 16S rRNA sequencing allowed us to identify a broader range of bacteria. Each technique seems to be able to extract bacterial genera in a similar way.

## 3. Discussion

Our study indicates that the High Pure PCR Template Preparation (Roche) kit preceded by both DTT and enzymatic digestion steps offers a higher DNA yield and more 16S rRNA gene real-time PCR extracted from sputum samples in comparison to the other evaluated conditions (Figure 3). Furthermore, no significant differences in terms of alpha-diversity (i.e., Shannon, Simpson and richness indices) have been detected across the different methods evaluated (Figure 5). 

A previous pilot study showed that no negative effect derived from the use of enzymes, DTT and different kits was identified. Variables were evaluated simultaneously, but a single variable approach is needed in order to have a deep understanding of single variables’ contributions [13]. For this reason, a consecutive approach has been adopted for the present study in order to understand the unique contribution of three different interventions: the use of DTT, the introduction of an enzymatic step and the choice of the best DNA extraction kit. 

The reactive sulphydryl groups of DTT have a mucolytic function and are used to reduce mucoprotein disulfide bonds. DTT is commonly used in standard microbiology to homogenize sputum samples, enhancing the isolation of microorganisms, and has been also adopted in different studies for microbiota analysis [14,15]. Sputum heterogeneity is one of the most challenging aspects of bacterial DNA recovery, especially in patients with CF and bronchiectasis. We evaluated the use of DTT through 16s rRNA real-time PCR in terms of (1) median levels, in order to understand if the presence of DTT was increasing bacterial DNA recovery; and (2) the coefficient of variation between replicates, in order to understand if the presence of DTT was able to better homogenize sputum and increase data reproducibility (Figure 1). The use of a reducing agent to treat this matrix allows the release of those bacteria entrapped in sputum plugs. We demonstrated that the addition of DTT increases the reproducibility of the data and facilitates sputum handling without affecting DNA extraction.

Lysostaphin and lysozyme have been extensively used in the literature to increase DNA recovery from Gram-positive bacteria [16,17,18]. These enzymes specifically lyse cell walls and ensure DNA recovery. Previously published experiences demonstrated clear advantages in adding lytic enzymes, including lysozyme and lysostaphin, to sputum samples. Cell wall destruction determined by the addition of these products improves real-time PCR detection and the sequencing of some Gram-positive bacteria, such as *S. aureus*, and this is evident already at low enzyme concentrations (lysostaphin at 0.18 mg/mL) [5]. Williamson and colleagues demonstrated the need to introduce these agents for microbiome analysis, even if there is no agreement in the literature on the concentration of lysostaphin needed to lyse bacteria in sputum samples. We demonstrated that sputum processing using these enzymes increases *S. aureus* DNA recovery, while the increase of lysostaphin concentrations seems not to affect *S. aureus* detection (Figure 2). Even if the presence of both pre-treatments will increase the time and costs of bacterial DNA extraction from sputum, the addition of this step seems to be needed in order to have more reliable and reproducible data.

We decided to use the Zymo Universal kit according to a previously performed pilot study [13] which compared different commercial kits across all conditions (with and without DTT and enzymes) in order to understand if combining/interfering effects were present among different variables. In this instance, we found no combined effect derived from the use of enzymes and DTT in terms of DNA yield, 16s rRNA gene and the *S. aureus* real-time PCR cycle threshold and microbiota analysis.

Studies evaluating respiratory microbiota in COPD and bronchiectasis patients used either BAL, spontaneous sputum or induced sputum [19,20,21,22]. DNA extraction methods across these experiences have been very heterogeneous, with some authors using chemical methods [14], others bead beating methods [23] and others a combination of physical lysis and methods such as phenol-chloroform [22]. The use of a combination of chemical and enzyme-based lysis increases DNA recovery from sputum samples. This method reduces the possibility of losing material after lysis because the whole of the lysate from samples is transferred into the DNA purification column. When bead-beating techniques are used, a fraction of lysate is retained in the beads phase after centrifugation. Even if Mobio, Zymobiomics and QIAGEN are based on mechanical disruption through bead beating, differences in methods could explain differences in extracted DNA yield. As reported in Table 1, Zymobiomics and QIAGEN also expose samples to enzymatic digestion in order to increase DNA extraction. Moreover, beads sizes differ between kits: QIAGEN and Mobio present glass beads of 0.1 mm of size, while Zymobiomics has beads of both 0.1 and 0.5 mm of size. Finally, column filters and reagents are specific for each kit. In terms of microbiota analysis, we were not able to identify statistically significant differences across the used kits, and we can therefore speculate that all the evaluated kits perform equally in representing analyzed samples (Figure 5 and Figure 6).

No similar studies have been previously designed and published to evaluate different DNA extraction methods from sputum to microbiota evaluations. Because of this, we were not able to define the correct sample size of our study a priori which should be interpreted as a pilot in its nature. Moreover, samples were tested in duplicate in real-time PCR (not triplicate) due to the low DNA yield of some samples. In the present study, we were not able to evaluate other methods for DNA extraction, including boiling, phenol-chloroform or automatic extraction, which have been previously reported in the literature, although the ones we have considered are those commercially available [14,22]. 

This is the first study able to evaluate different steps for DNA extraction from sputum for microbiota analysis in a sequential process which permits us to better control the impact of different interventions. The final evaluation consisted of the microbiota analysis of all the possible final conditions in order to have a general overview of the method. The use of blanks from each step and a positive control to increase the reliability of the sequencing techniques allowed us to exclude kits and environmental bacterial contamination.

## 4. Materials and Methods 

Three sequential experiments were performed to specifically address the study objectives. Sputum samples were collected from 32 adults with either CF or bronchiectasis referring to the Respiratory Department of Fondazione IRCCS Ca’ Granda Ospedale Maggiore Policlinico, Milan, Italy, between February and March 2018. Subjects signed an informed consent form and gave their approval to use their samples for the purpose of this study. Aliquots of sputum were collected and stored at −80 °C for all subsequent analyses, after which extraction samples were thawed and 0.1 g of sputum plug was selected for extractions.

Standard cultures for bacteria, fungi and non-tuberculous mycobacteria were performed by our local Cystic Fibrosis laboratory in accordance with the CF Foundation guidelines [24]. 100 microliters of the samples were plated on complete and selective media to isolate all potential pathogenic microorganisms. In the case of growth of potential pathogens, the colony morphotypes observed on the selective and non-selective media were identified by colonial morphology, pigment production or β-haemolysis, and then by biochemical assays and/or proteomic profiling by matrix assisted laser desorption-time of flight mass spectrometry (MALDI-TOF MS). After that, antibiotic susceptibility tests were performed by the Microscan WalkAway plus System (Beckman Coulter, Brea, CA, USA) [15].

**Experiment 1—DTT Treatment Evaluation.** In order to evaluate the need for sample homogenization, a total of 10 patients were enrolled in this experiment and 55 sputum samples were collected. Each sample was divided into two aliquots: the first aliquot was treated 1:1 with DTT (Sputafluid, Biolife, Italy) following the manufacturer’s instructions (DTT solution 10%, vortexed and left at room temperature for 15 min), while the second one received no treatment. After that, DNA was extracted in duplicate using Zymo Quick-DNA Universal Kit (Zymo, Irvine, CA, USA) according to the manufacturer’s instructions and eluted in 50 μL elution buffer. DNA extraction yield was measured through quantification by the Quant-iT dsDNA Assay Kit High Sensitivity and Qubit 3.0 Fluorometer (Invitrogen, Carlsbad, CA, USA). Subsequently, samples were diluted at 5 ng/µL to avoid the inhibition of PCR due to the high concentration of human DNA and tested through real-time PCR for SYBR Green for 16S rRNA gene amplification [25]. Each sample was tested in duplicate and the cycle threshold (*C*t) means between replicates, standard deviation and coefficient of variation were considered in order to identify potential differences between replicates. Endpoints for this experiment were real-time PCR for the 16S rRNA gene and coefficient of variation, as well as DNA yield and coefficient of variation. The DNA yield and 16S rRNA gene real-time PCR of the two groups identified as DTT-untreated and DTT are presented as a median with interquartile range (IQR). The coefficient of variation as the ratio of the standard deviation to the mean was calculated. The Mann–Whitney test was applied between groups. 

**Experiment 2—Lytic enzymes evaluation.** In order to understand if lysis using lysostaphin and lysozyme would increase sensitivity for specific genera, sputum samples from seven patients in which *S. aureus* had been isolated according to standard microbiology were selected and treated with DTT according to the results of Experiment 1. The enzymatic digestion was inserted in the procedure immediately after DTT treatment, carried out with lysozyme at 3.6 mg/mL and lysostaphin in two different conditions: 0.18 and 0.36 mg/mL (Sigma-Aldrich, Saint Louis, MO, USA). Samples were incubated at 37 °C for 30 min prior to DNA extraction. DNA extraction and DTT treatment were carried out as reported above. Real-time PCR for *S. aureus* was conducted on DNA extracts as already described [26]. Data were divided into three groups: (i) no lysostaphin; (ii) lysostaphin 0.18 mg/mL and (iii) lysostaphin 0.36 mg/mL, which are presented as medians (IQR). The endpoint included real-time PCR for *S. aureus*. Mann–Whitney and Kruskal–Wallis tests were applied to the three groups.

**Experiment 3—Evaluation of different kits for bacterial DNA extraction.** Fifteen samples were analyzed to understand which is the most suitable technique to extract DNA from sputum. Samples were pretreated according to Experiment 1 and 2 (DTT treatment and lysostaphin 0.18 mg/mL and lysozyme 3.6 mg/mL) and DNA was extracted using five different commercial kits: (1) Roche High Pure PCR Template Preparation Kit (Hoffmann, La Roche, Basel, Switzerland); (2) Zymo Quick-DNA Universal Kit (Zymo, Irvine, CA, USA); (3) MoBio PowerLyzer PowerSoil DNA isolation kit (Mobio, Loker Ave West, Carlsbad, CA, USA), actually sold by QIAGEN as the Qiagen DNeasy PowerSoil kit; (4) QIAGEN QIAmp Cador Pathogen Mini kit (Qiagen, Hilden, Germany); (5) ZymoBIOMICS DNA Miniprep Kit (Zymo, Irvine, CA, USA) (Table 1). The first two methods use the combination of both chemical and enzyme-based lysis, while the other three use a mechanical destruction through bead-beating. Commercial kits were used according to the manufacturer’s instructions and DNA was eluted in 50 μL elution buffer. DNA extraction was carried out as previously described, as well as real-time PCR targeting the 16s rRNA gene. The microbiota evaluation procedure is reported below. Negative controls from each DNA extraction kit and from PCR were also sequenced in order to also evaluate environmental contamination in kit reagents.

**Microbiota evaluation.** The V3-V4 variable regions of the 16S rRNA gene were amplified from DNA extracts using the 16S metagenomic sequencing library preparation protocol (Illumina, San Diego, CA, USA). PCR products, approximately sized 630 base pairs, were visualized using microfluidics-based gel electrophoresis on Bioanalyzer 2100 (Agilent, Santa Clara, CA, USA) and then were cleaned using AMPure XP magnetic bead-based purification (Beckman Coulter, Brea, CA, USA). Sample libraries were quantified using the Qubit as reported above and then pooled in an equimolar mode. Finally, the pool was sequenced on the MiSeq (Illumina, San Diego, CA, USA) sequencing platform, using a 2 × 300 cycle V3 kit and following standard Illumina sequencing protocols. 

**Bioinformatic and statistical analyses.** Demultiplexed paired-end reads in the FASTQ format were received from the Illumina MiSeq instrument. Sequencing data were processed following the UPARSE pipeline by Edgar [27], using USEARCH v10.0.240 (Tiburon, CA, USA) [28] and VSEARCH v2.3.4 (Oslo, Norway) [29]. Overall run quality was checked using FastQC v0.11.2 (Cambridge, UK) [30] and reports were summarized using MultiQC v1.4 (Stockholm, Sweden) [31]. Quality scores dropped towards the end of the reverse reads, so they were globally trimmed at position 275 before merging with the corresponding forward reads. Parameters for paired-end reads merging were set as follows: a minimum overlapping length of 95 base pairs, a minimum 90% identity of alignment, and the merged sequence lengths were restricted to 432–482 bases. Consensus sequences from all samples were pooled together and primers were stripped from both ends. This “raw” set of merged sequences was then quality-filtered and de-replicated to obtain a subset of high-quality unique sequences to be clustered into operational taxonomic units (OTUs). Sequences with more than one expected number of errors (EE) were discarded and singletons removed during de-replication. OTUs were clustered at a 97% identity threshold. The taxonomy prediction at the genus level for OTU sequences was performed via the SINTAX algorithm [32], using the RDP training set v16 as the reference database and 0.8 as confidence threshold. An OTU table was constructed by mapping the whole set of “raw” merged paired-end reads to the representative set of OTUs, using a 97% identity threshold. It was then filtered for low-abundance OTUs (<0.5 overall frequency), which were discarded, and normalized to the same number of reads per sample. This OTU table was used for all downstream analyses. Alpha diversity was measured for each sample using different metrics (Shannon entropy, Simpson estimators, and richness). These indices were then converted to the effective number of species (ENOS) [33] to be easily compared to each other. Results at the genus level were considered. Data were divided in five groups depending on the DNA extraction technique and presented as median (IQR). Sequences have been uploaded to the Sequence Read Archive (SRA) and are available under the project ID PRJNA488913. Endpoints included the DNA yield, real-time PCR for the 16S rRNA gene and alpha indices (Shannon and Simpson expressed as indices and as ENOS, and richness). Furthermore, a relative abundances comparison with standard microbiology was conducted. Mann–Whitney and Kruskal–Wallis tests were applied. A significance level of 0.05 was used for all the three experiments using GraphPad Prism 5 (La Jolla, CA, USA). 

## 5. Conclusions

In conclusion, the use of the Roche kit with the addition of a DTT treatment and enzymatic digestion with lysostaphin (0.18 mg/mL) and lysozyme offers better performance in terms of DNA yield and 16S rRNA gene real-time PCR extracted from sputum samples in comparison to other conditions. 

## Figures and Tables

**Table 1:** Comparison of different methods for DNA extraction (Information provided by manufacturer’s instructions and protocols).

Method	Basis of Function	Cost per Sample	Time per Sample (min)	Advantages	Disadvantages	Reference
Roche High Pure PCR Template Preparation Kit	Lysis Buffer and proteinase K digestion	2.62 €	16	Easy to use and quick protocol.	Milder extraction method than mechanical	Feigelman et al. 2017 [34]
Zymo Quick-DNA Universal Kit	Lysis Buffer and proteinase K digestion	2.49 €	18	Easy to use and quick protocol	Milder extraction method than mechanical	Blow et al. 2017 [35]
MoBio PowerLyzer PowerSoil DNA isolation kit	Bead beating	6.16 €	41	Possibility to lyse hard-to-lyse bacteria	Time consuming, possibility of DNA loss because of high number of steps	Willner et al. 2012 [36]
QIAGEN QIAmp Cador Pathogen Mini kit	Bead beating and proteinase K digestion	4.6 €	20	Possibility to lyse hard-to-lyse bacteria. Possibility to combine different methods.	Possibility of DNA loss	Hart et al. 2015 [37]
ZymoBIOMICS DNA Miniprep Kit	Bead beating and proteinase K digestion	5.12 €	63	Combination of enzymatic and mechanical distruption. Option to use different protocols	Time consuming, possibility of DNA loss because of high number of steps	Sohrabi et al. 2016 [38]
